# Dramatic and early response to low-dose steroid in the treatment of acute eosinophilic myocarditis: a case report

**DOI:** 10.1186/s12872-017-0547-9

**Published:** 2017-05-08

**Authors:** Yu-Wei Chen, Yu-Cheng Chang, Chieh-Shou Su, Wei-Chun Chang, Wen-Lieng Lee, Chih-Hung Lai

**Affiliations:** 1 Division of Cardiology, Department of Internal Medicine, Taichung Veterans General Hospital Chiayi Branch, Chiayi, Taiwan, Republic of China; 2 Department of Cardiology, Asia University Hospital, Taichung, Taiwan, Republic of China; 3 Division of Interventional Cardiology, Cardiovascular Center, Taichung Veterans General Hospital, 1650 Taiwan Boulevard Sect. 4, Taichung, 40705 Taiwan, Republic of China; 4 School of Medicine, National Yang-Ming University, Taipei, Taiwan, Republic of China

**Keywords:** Case report, Eosinophilia, Heart failure, Hypereosinophilic syndrome, Left ventricular hypertrophy, Myocarditis, Steroids

## Abstract

**Background:**

Eosinophilic myocarditis encompasses a variety of etiologies and the prognosis varies. For patients with a hypersensitive response to medications, high-dose corticosteroids and discontinuation of culprit medications are the main treatments.

**Case presentation:**

We reported a young man with biopsy-proven eosinophilic myocarditis which was possibly induced by Chinese herbal medicine. His heart failure and left ventricular hypertrophy improved soon after low-dose corticosteroid.

**Conclusion:**

Low-dose corticosteroid may be effective in selected patients with eosinophilic myocarditis. Early echocardiographic follow-up is mandatory for evaluation of the clinical response.

**Electronic supplementary material:**

The online version of this article (doi:10.1186/s12872-017-0547-9) contains supplementary material, which is available to authorized users.

## Background

Eosinophilic myocarditis is a relatively rare disease, and an early diagnosis is crucial to obtain good outcomes. Three different stages of the disease have been identified: acute necrotic stage, thrombus formation stage, and fibrotic stage. However, the clinical presentation varies widely, including pericarditis, acute coronary syndrome, heart failure, cardiogenic shock, and aneurysm formation [1–3]. High-dose corticosteroid therapy is considered as first-line treatment in many studies [4–6]. However, the efficacy remains controversial and the optimal dosage and treatment duration remain uncertain [7].

## Case presentation

**Fig. 1:**
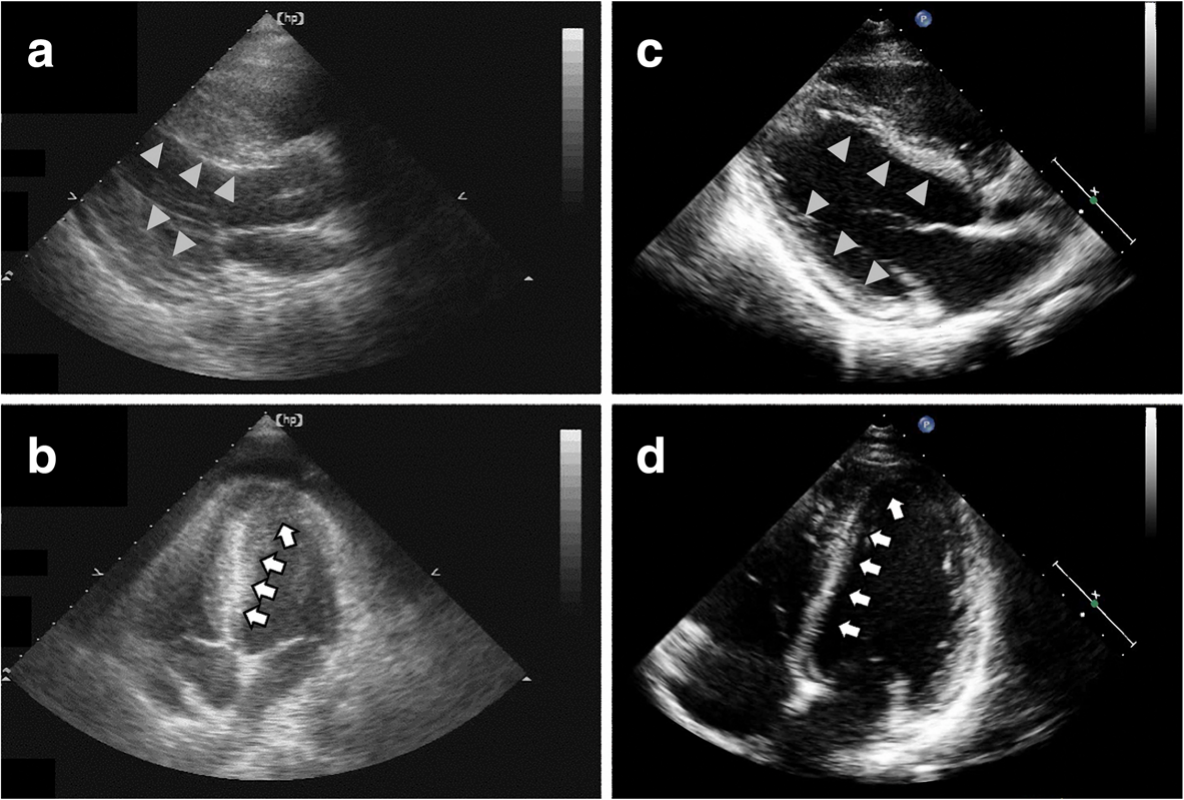
Transthoracic echocardiography showed dramatic improvement of left ventricle wall thickness and systolic motion after prednisolone treatment (10 mg) for 3 days. **a** Parasternal long axis view before treatment showed increased wall thickness of the septum and posterior wall (*arrowheads*). **b** Apical four chamber view before treatment showed increased wall thickness of the inferior septum, anterior lateral wall and apex (*arrows*). **c** Parasternal long axis view after treatment showed resolution of thickened wall of the septum and posterior wall (*arrowheads*). **d** Apical four chamber view after treatment showed resolution of the thickened inferior septum, anterior lateral wall and apex (*arrows*)



A 31-year-old man, with longstanding allergic rhinitis and asthma, presented with facial edema, chest tightness and dyspnea on exertion for 1 week. He had experienced one episode of generalized urticaria about 1 month prior, which subsided after he took an unknown Chinese herbal medicine for 2 weeks. The physical examination was unremarkable. Laboratory tests showed an increased white blood cell count (13,880/mL) with 67.7% neutrophils, 15.9% lymphocytes and 8.5% eosinophils (absolute eosinophil counts 1180/mL), and increased levels of C-reactive protein (2.31 mg/dL, normal range < 0.3 mg/dL), creatine phosphokinase (CPK, 398 U/L, normal range 10–160 U/L) and troponin-I (2.52 ng/mL, normal range < 0.034 ng/mL). Chest radiography revealed mild cardiomegaly and electrocardiogram (ECG) revealed sinus tachycardia, left axis deviation, poor R wave progression, and diffuse Q waves in the inferior and precordial leads. Transthoracic echocardiography (TTE) showed left ventricle (LV) wall thickening (septum/posterior wall: 2.0 cm/1.4 cm) with mild systolic dysfunction (LV ejection fraction: 46%) (Fig. 1a and b; An additional movie file shows this in more detail [see Additional file 1]). Based on the suspicion of myocardial infarction, cardiac angiography was done and revealed non-stenotic coronary arteries, and the left ventriculography showed mild systolic dysfunction.

**Additional file 1: Movie 1.** Was echocardiography in left ventricular long axis view before treatment, which showed systolic dysfunction with increased septum and posterior wall thickness. (AVI 1004 kb)


**Fig. 2:**
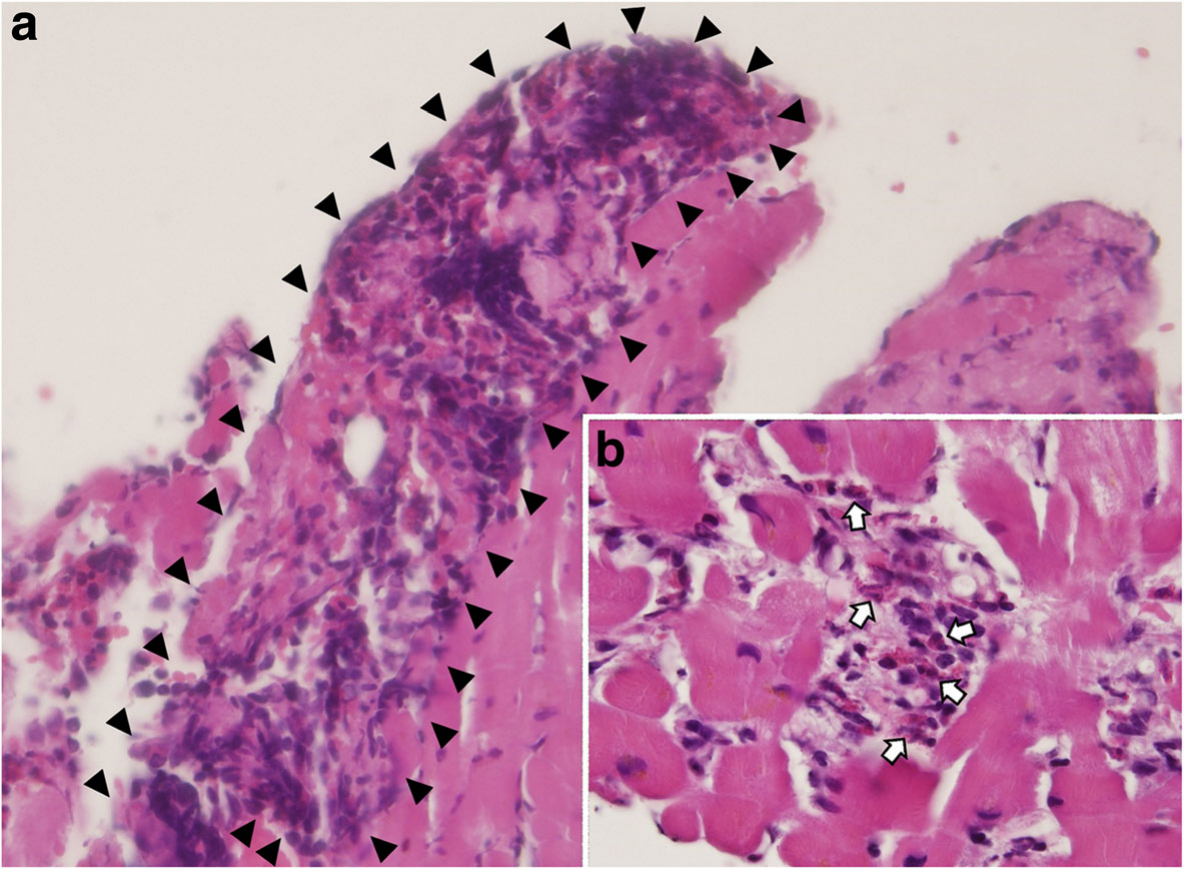
Endomyocardial biopsy specimen revealed myocarditis with increased eosinophils infiltration, consistent with eosinophilic myocarditis. **a** The specimen showed extensive inflammatory cell infiltration (*arrowheads*), composed of mononuclear lymphocytes and numerous eosinophils and some fibrosis (H&E stain, × 400). **b** The specimen showed diffuse eosinophils infiltration (*arrows*, H&E stain, × 1000)

Under the impression of acute myocarditis, right ventricular endomyocardial biopsy was performed. Histopathological examination revealed myocarditis with increased eosinophilic infiltration (Fig. 2), compatible with eosinophilic myocarditis. Follow-up laboratory test showed a moderate increase of absolute eosinophil counts up to 2577/mL (white blood cell count 9910/mL with 26.0% eosinophils). On the other hand, serum CPK decreased to 291 U/L. Follow-up ECG revealed normalization of R wave progression and disappearance of the Q waves in the precordial leads.

To rule out other etiologies of hypereosinophilic syndrome, several tests were performed. Bone marrow biopsy, testing for the lymphoma phenotype and *FIP1L1-PDGFRA* gene mutations, and abdominal computed tomography (CT) all revealed no evidence of solitary or hematological malignancies. Testing for serum antinuclear antibodies was negative, and the patient did not meet the diagnostic criteria for immunological diseases.

Based on the patient’s medical course and testing results, acute eosinophilic myocarditis caused by a hypersensitive drug reaction was diagnosed. The patient was treated with low dose prednisolone (10 mg per day), combined with angiotensin-converting-enzyme inhibitors and beta-blockers. Three days after beginning treatment, follow-up TTE showed dramatic improvement of LV wall thickening (septum/posterior wall: 1.1 cm/1.1 cm) with complete recovery of systolic function (LV ejection fraction: 61%) (Fig. 1c and d; An additional movie file shows this in more detail [see Additional file 2]). In addition, the peripheral blood eosinophil count decreased to normal range (absolute eosinophil count 273/mL). Because of the improvement in symptoms, eosinophil count, and LV hypertrophy in echocardiography, we tapered of the prednisolone to 5 mg QD 1 month later, and then discontinued prednisolone after another 2 months. After 6 months of follow-up, the patient was symptom free and did not received any further steroid treatment.



**Additional file 2: Movie 2.** Was echocardiography in left ventricular long axis view after treatment, which showed full recovery of systolic function with normal wall thickness in the septum and posterior wall. (AVI 2595 kb)


## Discussion

In the early stage of eosinophilic myocarditis, the clinical presentation may be subtle and easily ignored by clinicians. Some patients may initially have a normal peripheral eosinophil count, which makes the diagnosis even more difficult [8, 9]. In a case series from Korea, 25% of patients with acute myocarditis who initially had a normal eosinophil count were proven to have eosinophilic myocarditis by endomyocardial biopsy [10]. Therefore, timely awareness and early endomyocardial biopsy is mandatory for a prompt diagnosis. Our patient had initially only a mildly elevated eosinophil count at presentation, and the diagnosis of eosinophilic endocarditis was made by early endomyocardial biopsy. Currently, an endomyocardial biopsy is considered to be the gold standard for the diagnosis of eosinophilic myocarditis [11–15].

The underlying causes of eosinophilic myocarditis are various, including hypereosinophilic syndrome, malignancies, parasite infection, autoimmune diseases, drug reactions, allergy and transplant rejection [7, 16, 17]. Our patient had a history of allergic rhinitis and asthma, and one episode of generalized urticaria. The most likely etiology in our case was a hypersensitive response to the Chinese herbal medicine that he had taken about 2 weeks prior. Nevertheless, we performed a complete work-up to exclude other etiologies. Furthermore, these negative results of the testing allowed for a more convincing diagnosis of hypersensitivity myocarditis. However, the cost of serial surveys and the invasiveness of a bone marrow biopsy should be balanced by their diagnostic value. Further studies should focus on the predictive value of each screening tool to guide clinicians in choosing the best algorithm to survey the possible etiology of eosinophilic myocarditis.

For eosinophilic myocarditis, there are many different imaging approaches for diagnosis and disease follow-up. One recently published review compared the roles of echocardiography, cardiac magnetic resonance imaging (MRI), cardiac CT and position emission tomography (PET)-CT. Echocardiography is a first-line method for diagnosis and further follow-up, whereas cardiac MRI is the gold standard noninvasive method for diagnosis of myocarditis. Cardiac CT and PET-CT are useful to exclude significant coronary artery disease and other etiologies of hypereosinophilia, such as autoimmune diseases [18]. In our patient, we chose echocardiography for initial diagnosis and monitoring treatment response. Cardiac MRI was not needed because we performed early endomyocardial biopsy, which was diagnostic for eosinophilic myocarditis. Furthermore, cardiac CT and PET-CT were not necessary as other etiologies were rule-out by the other testing performed.

Our patient had a good response to low-dose prednisolone (10 mg per day). This dosage is relatively low compared to that used in previous studies regarding the treatment of hypersensitivity myocarditis, in which the dosage of corticosteroids ranged from prednisolone 1 mg/kg per day to methylprednisolone 500 mg, or 1 g per day for 3 days, followed by tapering [2, 4–6]. There were 4 reasons we choose to use a low dose in our patient. First, the patient was young and healthy, without a history of any systemic diseases. Second, the most possible etiology was hypersensitivity. When hypersensitivity is suspected, it is of utmost importance to discontinue any possible medication instead of immediate administration of high-dose corticosteroids. Third, the clinical presentation was relatively mild, including early improvement of serum CPK and ECG changes. Last but not least, echocardiography was used to monitor the treatment response, and improvement was seen after 3 days of treatment. Had a deterioration of his condition occurred, higher dose corticosteroids would have been started.

Our patient responded well to lower steroid dosage. One possible explanation is that hypersensitivity myocarditis encompasses a wide variety of hypersensitivity responses to different drugs or substances, and exposure times, which can lead to differences in severity and therapeutic response. Our patient had relatively less severe disease, which contributed to the prompt response to low-dose prednisolone and withdrawal of the drug which may have invoked the response. Some studies have shown improvements in LV systolic wall motion and wall thickness without the use of corticosteroid, which may support our finding [19, 20]. In addition, his clinical condition and echocardiography finding improved after only 3 days of corticosteroid treatment, which is also shorter compared to previous studies [9, 21]. This may also be explained by milder disease with early resolution of interstitial edema in our patient. In our opinion, a rapid response to steroid therapy may suggest mild disease and a good prognosis. In addition, we suggest that close follow-up with echocardiography is a useful tool to monitor response. Rapid improvements in wall thickening and systolic function may mean a good prognosis.

## Conclusion

Eosinophilic myocarditis with a possible etiology of hypersensitivity response and mild clinical presentation may be initially treated with low-lose corticosteroid. At the same time, close monitoring of treatment response with echocardiography should be considered.

## Abbreviations

LV: Left ventricular
ECG: Electrocardiogram
TTE: Transthoracic echocardiography
CPK: Creatine phosphokinase
CT: Computed tomography
MRI: Magnetic resonance imaging
PET: Position emission tomography

## References

[1] Carrilho-Ferreira P, Silva Marques J, Gouveia R, Brito D (2014). Eosinophilic myocarditis with left ventricular apical aneurysm. Eur Heart J Cardiovasc Imaging.

[2] Al Ali AM, Straatman LP, Allard MF, Ignaszewski AP (2006). Eosinophilic myocarditis: case series and review of literature. Can J Cardiol.

[3] Li Q, Gupta D, Schroth G, Loghin C, Letsou GV, Buja LM (2002). Images in cardiovascular medicine. Eosinophilic Pericarditis Myocarditis. Circulation.

[4] Getz MA, Subramanian R, Logemann T, Ballantyne F (1991). Acute necrotizing eosinophilic myocarditis as a manifestation of severe hypersensitivity myocarditis. Antemortem diagnosis and successful treatment. Ann Intern Med.

[5] Adsett M, West MJ, Galbraith A, Duhig E, Lange A, Palka P (2003). Eosinophilic heart: marked left ventricular wall thickening and myocardial dysfunction improving with corticosteroid therapy. Echocardiography.

[6] Li H, Dai Z, Wang B, Huang W (2015). A case report of eosinophilic myocarditis and a review of the relevant literature. BMC Cardiovasc Disord.

[7] Seguela PE, Iriart X, Acar P, Montaudon M, Roudaut R, Thambo JB (2015). Eosinophilic cardiac disease: Molecular, clinical and imaging aspects. Arch Cardiovasc Dis.

[8] Morimoto S, Kubo N, Hiramitsu S, Uemura A, Ohtsuki M, Kato S (2003). Changes in the peripheral eosinophil count in patients with acute eosinophilic myocarditis. Heart Vessel.

[9] Fozing T, Zouri N, Tost A, Breit R, Seeck G, Koch C (2014). Management of a patient with eosinophilic myocarditis and normal peripheral eosinophil count: case report and literature review. Circ Heart Fail.

[10] Youn JC, Shim HS, Lee JS, Ji AY, Oh J, Hong N (2014). Detailed pathologic evaluation on endomyocardial biopsy provides long-term prognostic information in patients with acute myocarditis. Cardiovasc Pathol.

[11] Caforio AL, Pankuweit S, Arbustini E, Basso C, Gimeno-Blanes J, Felix SB (2013). Current state of knowledge on aetiology, diagnosis, management, and therapy of myocarditis: a position statement of the European Society of Cardiology Working Group on Myocardial and Pericardial Diseases. Eur Heart J.

[12] Leone O, Veinot JP, Angelini A, Baandrup UT, Basso C, Berry G (2012). 2011 consensus statement on endomyocardial biopsy from the Association for European Cardiovascular Pathology and the Society for Cardiovascular Pathology. Cardiovasc Pathol.

[13] Kindermann I, Barth C, Mahfoud F, Ukena C, Lenski M, Yilmaz A (2012). Update on Myocarditis. J Am Coll Cardiol.

[14] Group JJW (2011). Guidelines for diagnosis and treatment of myocarditis (JCS 2009): digest version. Circ J.

[15] Cooper LT, Baughman KL, Feldman AM, Frustaci A, Jessup M, Kuhl U (2007). The role of endomyocardial biopsy in the management of cardiovascular disease: a scientific statement from the American Heart Association, the American College of Cardiology, and the European Society of Cardiology. Circulation.

[16] Valent P, Klion AD, Horny HP, Roufosse F, Gotlib J, Weller PF (2012). Contemporary consensus proposal on criteria and classification of eosinophilic disorders and related syndromes. J Allergy Clin Immunol.

[17] Sagar S, Liu PP, Cooper LT (2012). Jr Myocarditis Lancet.

[18] Kuchynka P, Palecek T, Masek M, Cerny V, Lambert L, Vitkova I (2016). Current Diagnostic and Therapeutic Aspects of Eosinophilic Myocarditis. Biomed Res Int.

[19] Yanagisawa T, Inomata T, Watanabe I, Maekawa E, Mizutani T, Shinagawa H (2011). Clinical significance of corticosteroid therapy for eosinophilic myocarditis. Int Heart J.

[20] Kazama R, Okura Y, Hoyano M, Toba K, Ochiai Y, Ishihara N (2003). Therapeutic role of pericardiocentesis for acute necrotizing eosinophilic myocarditis with cardiac tamponade. Mayo Clin Proc.

[21] Imran SS, Tan KB, Chee LS, Yang SC, Chew RW, Yee OH (2010). Eosinophilic myocarditis: an unusual presentation. J Am Coll Cardiol.

